# Maternal and neonatal colonisation of group B streptococcus at Muhimbili National Hospital in Dar es Salaam, Tanzania: prevalence, risk factors and antimicrobial resistance

**DOI:** 10.1186/1471-2458-9-437

**Published:** 2009-12-01

**Authors:** Agricola Joachim, Mecky I Matee, Furaha A Massawe, Eligius F Lyamuya

**Affiliations:** 1 Department of Microbiology and Immunology, School of Medicine, Muhimbili University of Health and Allied Sciences, P.O. Box 65001, Dar es Salaam, Tanzania; 2 Department of Gynaecology and Obstetrics, School of Medicine, Muhimbili University of Health and Allied Sciences, P.O. Box 65001, Dar es Salaam, Tanzania

## Abstract

**Background:**

Group B streptococcus (GBS), which asymptomatically colonises the vaginal and rectal areas of women, is the leading cause of septicemia, meningitis and pneumonia in neonates. In Tanzania no studies have been done on GBS colonisation of pregnant women and neonates. This study was conducted in Dar es Salaam, Tanzania to determine the prevalence of GBS colonisation among pregnant women, the neonatal colonisation rate and the antimicrobial susceptibility, thus providing essential information to formulate a policy for treatment and prevention regarding perinatal GBS diseases.

**Methods:**

This cross sectional study involved 300 pregnant women attending antenatal clinic and their newborns delivered at Muhimbili National Hospital (MNH) between October 2008 and March 2009. High vaginal, rectal, nasal, ear and umbilical swabs were cultured on Todd Hewitt Broth and in 5% sheep blood agar followed by identification of isolates using conventional methods and testing for their susceptibility to antimicrobial agents using the Kirby-Bauer method.

**Results:**

GBS colonisation was confirmed in 23% of pregnant women and 8.9% of neonates. A higher proportion of GBS were isolated from the vagina (12.3%) as compared to the rectum (5%). Prolonged duration of labour (>12 hrs) was significantly shown to influence GBS colonisation in neonates P < 0.05. Other risk factors such as prolonged rupture of membrane, intrapartum fever, low birth weight and HIV infection did not correlate with GBS colonisation. All isolates were sensitive to vancomycin and ampicillin. Resistance to clindamycin, erythromycin and penicillin G was found to 17.6%, 13% and 9.4%, respectively.

**Conclusion:**

Our findings seem to suggest that a quarter of pregnant women attending ANC clinic at MNH and approximately 10% of their newborns are colonised with GBS. All isolates were found to be sensitive to vancomycin and ampicillin which seem to be the most effective antibiotics for the time being. However there is a need for continuous antibiotics surveillance of GBS to monitor trend of resistance. The high isolation frequency of GBS among pregnant women suggests routine antenatal screening at 35 to 37 weeks of gestation in order to provide antibiotic prophylaxis to GBS carrier.

## Background

Group B Streptococcus (GBS) is now recognized to be an important cause of maternal and neonatal morbidity and mortality in many parts of the world [1,2]. GBS infections tend to occur more commonly among adults than in neonates, but the overall mortality is higher in neonates [3]. Risk of disease is greater in pregnant women than in men and non-pregnant women. At birth, infants who are born to colonized mothers may also become colonised on their mucosal surfaces such as oral, nasopharynx, vaginal and anal mucosa and skin [4]. Approximately 60% of infants born to colonised mother become colonised with their mother's organisms [3]. The likelihood of neonatal colonisation at birth is higher if the mother is heavily colonised [3].

GBS infection is the leading cause of perinatal bacterial infection [5], being commonly responsible for septicemia, meningitis and pneumonia in neonates. However, the burden of perinatal GBS disease varies between countries. Intrapartum antimicrobial prophylaxis to GBS carriers has been reported to be effective in reducing GBS disease. Prevention guidelines for perinatal GBS disease were issued by Centers for Disease Control and Prevention (CDC) in 1996, and revised in 2002 [6,7]. Since its introduction, the incidence of early-onset neonatal infections has decreased by 62% [7].

In Tanzania, as is the case in several other sub-Saharan countries in Africa, the rate of GBS colonisation among pregnant women and neonates has not been studied and therefore to date no strategies have been formulated to prevent neonatal GBS infection in the country. Thus the aims of this study was to determine the prevalence and risk factors of maternal and neonatal colonisation with GBS at Muhumbili National Hospital (MNH), Dar es Salaam Tanzania in order to generate local data that will inform the development of rational interventions for GBS infection and disease.

## Methods

### Study design and setting

This was a hospital based cross-sectional study conducted at Muhimbili National Hospital (MNH), Dar es Salaam, Tanzania between October 2008 and March 2009. MNH is a tertiary facility which handles referrals from peripheral and upcountry hospitals.

### Study population

The study included 300 third trimester pregnant women (from 37 weeks of gestation) attending the antenatal clinics at MNH for routine antenatal visits and their neonates (180) delivered at the MNH labor ward. Sample size was calculated from the following formula n = z2p (100-p)/e^2 ^where p, in the absence of local data, was taken as the median prevalence of GBS carriage among pregnant women in African countries (16%) [8,9]. All consenting mothers with gestation age ≥ 37 weeks were included. Participants with history of using antibiotic(s) within two weeks prior to recruitment were excluded from the study.

### Data collection

Data was collected after obtaining written informed consent using a standard structured questionnaire designed to obtain social demographic data and other relevant information such as maternal age, gestational age, previous obstetric history, history of current pregnancy, parity, marital status, mode of delivery, administration of antibiotics during labor and neonate's birth weight. Pregnant women were interviewed at 37 weeks of gestation, during labour and after delivery.

### Specimen collection and transport

High vaginal and rectal swabs were collected at 37 weeks of gestation using sterile swab stick. In addition, umbilical swabs, ear canal and nasal swabs were collected from neonates within one hour after delivery. The swabs were inoculated directly into Todd Hewitt Broth (Oxoid Ltd) and transported to the central pathology laboratory of the MNH for processing within 2 hours of collection. About three millilitres of venous blood were drawn aseptically into sterile vacutainer tubes (BD NJ USA) without anticoagulant for detection of HIV antibodies and in EDTA (BD NJ USA) vacutainer tubes for quantification of CD4+T lymphocytes counts.

### Laboratory Procedures

The swabs were inoculated in Todd-Hewitt broth (Oxoid Ltd) containing nalidixic acid (15 mg/L) and gentamicin (8 mg/L) and incubated at 37°C in 5% Co_2 _for 24 h. Subcultures were performed in 5% sheep blood agar for isolation of GBS. Broth cultures showing no visible turbidity after overnight incubation were further re-incubated and then sub cultured after 48 hours on sheep blood agar. Presumptive identification of GBS was made by traditional physiological and biochemical methods. These included Gram stain, catalase reaction, hemolytic activity on sheep blood agar plates, hippurate and CAMP tests. Confirmative identification of Group B Streptococcus was done using Streptex- agglutination test (Remel Europe Ltd). Finally, antimicrobial susceptibility of all GBS isolates was determined by Kirby-Bauer disk diffusion method according to Clinical Laboratory Standard Institute (formerly NCCLS). The antimicrobials included; penicillin G (10 U), ampicillin (10 μg), clindamycin (2 μg.), erythromycin (15 μg), vancomycin (30 μg), ciprofloxacin (5 μg), ceftriaxone (30 μg) and co-trimoxazole 25 μg. *S. agalactiae *ATCC 27956 was used as a control organism.

### HIV screening

HIV testing was done using Tanzania's National HIV rapid test algorithm [10]. SD-Bioline HIV 1/2 (SD Standard Diagnostic, Inc Korea) was used for initial screening and reactive samples were confirmed by Determine™ (Abbott Laboratory, Tokyo Japan). A sample was considered seronegative if it was non reactive at screening and seropositive when reactive on both assays. There were no discordant samples.

### CD4+ T lymphocytes enumeration

This was determined by using Becton Dickson FACS Calibur flow cytometry.

### Data analysis

Data was analysed using SPSS for windows version 13.0 [11]. Comparison of proportions of maternal and neonatal colonisation of GBS and statistical significance of GBS in HIV infected and non HIV infected pregnant women colonised with GBS were tested by using the Pearson's Chi-squared test (χ^2^). Where the numbers in a cell was less than five, a Fisher's exact test was used. P-values ≤ 0.05 were considered statistically significant.

### Ethical issues

Ethical and research clearance was obtained from Muhimbili University of Health and Allied Sciences (MUHAS) Senate Research and Publication Committee. A written informed consent was obtained from each participant prior to enrolment. HIV- pre and post test counselling were provided to the participants according to the Tanzania national guidelines for HIV testing and counselling [12]. Any other requested additional information was provided to participants by study personnel. HIV positive women were offered prevention of mother to child transmission (PMTCT) services and also referred to HIV care and treatment clinic within MNH for further management, according to the National guidelines for Care and Treatment, Ministry of Health, Tanzania. GBS results of neonates were relayed to attending doctors for their management. All participants' results were kept confidentially.

## Results

Three hundred pregnant women (from 37 weeks of gestation) and 180 neonates were enrolled in the study between October and March 2009. There was loss of follow up of 120 neonates, due to the fact that not all pregnant women delivered at MNH. GBS were isolated from 69 (23%) pregnant women and from16 (8.9%) neonates. Vaginal carriage rate was higher 37 (12.3%) than rectal colonisation rate 15(5%). The proportion of GBS isolated from both sites was 17(5.7%).

Table 1 summarises the rate of GBS colonisation by social demographic characteristics. The age of the women ranged from 16-44 years with mean age of 26.6 years (SD ± 5.1). Generally, GBS colonisation did not appear to be influenced by maternal age, marital status, education level or number of sexual partners. The study revealed a higher colonisation rate among the age group 30-34 years (32.1%) but lower (15.4%) in women aged less than 20 years, however the difference was not statistically significant (P > 0.05). GBS colonisation was also more prevalent among women with no formal education when compared with those with secondary education, post secondary and primary education however this was not statistically significant (P > 0.05). Of the 300 women investigated, the majority (93%) reported having one sexual partner while 21(7%) had multiple sexual partners in the last one year preceding the study. GBS colonisation did not differ significantly with marital status, being 33.3% among divorced women, 23.8% among married and 19.3% among single women (P > 0.05).

**Table 1:** Association between social demographic factors and GBS colonisation among pregnant women

Variable	Total	Number of GBS isolated	Percentages	P-value
Age group				
<20	26	4	15.4	
20-24	86	19	22.1	
25-29	107	23	21.5	0.43
30-34	56	18	32.1	
>35	25	5	20	

Marital status				
Single	52	10	19.2	
Married/Cohabiting	244	58	23.8	0.74
Divorced	3	1	33.3	
Widow	1	0	0	

Education level				
None	8	3	37.5	
Primary	145	30	20.7	0.54
Secondary	104	28	26.0	
Post secondary	43	8	20.9	

Number of sexual				
partner One	279	65	23.3	0.65
Multiple	21	4	19	

Table 2 shows maternal obstetric factors and HIV status in relation to GBS colonisation. Women with gestation age between 41 and 42 weeks were found to have significantly higher colonisation rate (46.7%), followed by those with gestational ages of 37-38 weeks (23.6%) and 39-40 weeks (15.4%), P < 0.05. The parity ranged from zero to seven with parity mean of 0.88. Colonisation rate was higher (50%) in women who had delivered four or more times and lower in women who had delivered once (19.8%). However, this difference was not statistically significant (P > 0.05).

**Table 2:** Association between obstetric factors, HIV infection and GBS colonisation among pregnant women n = 300

Variable	Total	Number GBS isolated	Percentages	P-value
Gestational age				
37-38	220	52	23.6	
39-40	65	10	15.4	0.03
41-42	15	7	46.7	

Parity				
0	138	33	23.9	
1	91	18	19.8	
2	51	11	21.6	0.45
3	12	3	25	
4	6	3	50	
≥ 5	2	1	50	

Previous obstetric history				
Normal healthy baby	137	32	23.2	0.44
Premature	6	0	0	
Spontaneous abortion	18	2	11.1	
Stillbirth	14	4	28.6	

Dysuria				
No	273	64	23.4	
Yes	27	5	18.5	0.38

Vaginal discharge				
No	284	66	23.2	0.67
Yes	16	3	18.8	

HIV infection				
No	276	67	24.3	0.08
Yes	24	2	8.3	

*125 out of 300 pregnant women were primigravida and had no previous obstetric history.

Women with previous history of stillbirth had higher rate (28.6%) of GBS colonisation followed by those with normal previous obstetric history (23.2%) and with history of spontaneous abortion (11.1%) (P > 0.05). No GBS colonisation was observed in women with previous history of premature labour. Sixty four (23.4%) of the women with GBS colonisation had no history of dysuria. In this study urine culture was not done in patients with dysuria.

HIV infection was detected in 24/300 (8%) of the study population, among whom 2/24 (8.3%) had GBS colonisation (P > 0.05) and one had CD4 T-cell count less than 200 cells/μL and the other had CD4 T-cell count above 200 cells/μl.

The association between GBS colonisation rate in neonates and maternal obstetric factors is summarized in Table 3. Prolonged duration of labour was significantly shown to influence GBS colonisation in neonates; 6/17 (35.3%), P < 0.001. The rate of GBS colonisation was higher 14/139 (10.1%) in neonates born by spontaneous vaginal delivery compared to 2/41 (4.9%) among neonates born by caesarean section, P > 0.05 however, was not statistically significant. Nearly all women (15/16) who had history of taking antibiotic during labour had neonates with no GBS colonisation. The time taken between antibiotic intake and delivery was approximately one to four hours for those who were given intravenous antibiotics (ampicillin or ceftriaxone) at labour ward. The reasons for antibiotic administration included; prolonged duration of labor with fetal distress, caesarean section, and intrapartum fever.

**Table 3:** Association between obstetric factors and GBS colonisation rate in neonates (n = 180)

Variable	Total	Number GBS isolated	Percentages	P-value
Mode of delivery Vaginal delivery	139	14	10.1	
Caesarean	41	2	4.9	0.61

Time of membrane rupture	178	16	9	0.65
≤ 18 hrs	2	0	0	
>18 hrs				

Intrapartum fever				
No	177	16	9	0.58
Yes	3	0	0	

Antibiotic use before/during labour				
No	164	15	9.1	0.69
Yes	16	1	6.3	

Duration of labour				
≤ 12 hrs	163	10	6.1	0.00
>12 hrs	17	6	35.3	

Weight of baby				
<2.5 kg	20	1	5	0.51
≥ 2.5 kg	160	15	9.4	

Table 4 presents the antimicrobial susceptibility pattern of GBS isolated from different sites. The GBS isolates were all (100%) sensitive to vancomycin and ampicillin, (90%-100%) sensitive to penicillin G and ciprofloxacin and (80-90%) sensitive to clindamycin, erythromycin, cotrimoxazole and ceftriaxone.

**Table 4:** Antimicrobial Sensitivity pattern of GBS isolated from pregnant women and neonates

Antimicrobial agent	GBS isolation (% of sensitivity)		
	
	Vaginal	Rectal	Neonates
Penicillin G	98.1	90.6	100

Ampicillin	100	100	100

Vancomycin	100	100	100

Erythromycin	87.0	81.3	81.3

Clindamycin	83.3	84.3	87.5

Ciprofloxacin	94.4	93.8	100

Cotrimoxazole	90.7	84.4	87.5

Cefriaxone	87.0	84.4	93.8

## Discussion

This study at the MNH in Dar es Salaam, Tanzania shows the overall prevalence of GBS among pregnant women and neonates to be 23% and 8.9%, respectively. These findings are consistent with reports from other developing countries [8,13], but lower than those reported in Zimbabwe 31.6% and Trinidad 32.9% [9,14], indicating significant country variations [15]. The variations between countries could possibly be due, at least in part, to differences in sampling sites and techniques. For instance, in this study due to investigating sexually transmitted infections, we used high vaginal swabs which is in keeping with some previous studies [9,16], while others investigators have used lower third of vaginal site [5,6]. Other variations in isolation frequency could be due differences in culture methods, and type of culture media used as well as populations investigated [4].

In this study vaginal carriage rate (12.3%) was higher than rectal colonisation rate (5%), which is comparable to a study done in Zimbabwe by Moyo et al [13] in which vaginal and rectal colonisation rates were 12.6% and 6.3%, respectively. The fact that GBS was at times isolated from one and not the other site clearly indicate that it is important to sample both vagina and rectum when screening for GBS carriage in pregnant women.

Notably, there was loss of 120 neonates to follow up due to the fact that mothers delivered in other facilities. One twenty neonates made 40% of neonates and this could have affected the reported neonatal colonisation rate.

Samples cultured from mothers and neonates showed that 37% of the pairs had positive GBS culture results, which is in keeping with other studies reporting transmission rates ranging from 29% to 85% [17,18].

Of all the factors investigated, maternal colonisation was significantly higher in women of gestation age between 41 and 42 weeks compared to women of gestation age between 39 and 40 weeks, indicating an increase GBS carriage with gestation age. The other maternal factors including social demographic factors were not significantly associated with GBS colonisation.

In the present study GBS was isolated more frequently from women of age group 30-34 (32.1%) compared with women aged <20 years (15.4%), which is in contrast with reports from another study showing high isolation frequencies in women younger than 20 years [1]. These differences are difficult to explain but possibly underscore the fact that GBS colonisation might be influenced by multiple factors which may vary from one geographical location to another.

This study revealed that women with no formal education (34.8%) were more likely to be colonised with GBS, a finding that is consistent with observations by Regan et al [19]. The association could be partly explained by the difference in personal hygiene, which is more likely to be better among educated than the less educated women.

GBS colonisation in the studied population was not associated with either HIV infection or CD4+ T cell count, probably due to the small sample of HIV infected pregnant women among the studied population and the fact that only one woman had CD4 counts < 200 cells/μl.

Of all possible factors for GBS colonisation in neonates that were investigated only prolonged duration of labour showed significant association, P < 0.001, probably due to prolonged exposure of the neonate in the birth canal. The strong association between prolonged labour and GBS colonisation calls for routine antibiotic prophylaxis in such women in order to decrease the chances of subsequent neonatal infection.

Other risk factors such as prolonged rupture of membrane, intrapartum fever, mode of delivery and low birth weight did not influence GBS colonisation in neonates. The lack of association with these factors can possibly be explained by the fact that the numbers of participants in this study with such risk factors were small (2%). We did not investigate the timing of caesarean section in relation to prelabor and post labour rupture of membrane versus spontaneous vaginal delivery, which could be viewed as one of the limitations of the study. However, the small number of women who delivered by caesarean section may not show any potential association.

Notably all GBS isolated were sensitive to ampicillin and vancomycin, implying that these two antibiotics could be used for empiric prophylaxis. Most isolates (90% to 98%) were sensitive to ciprofloxacin and penicillin G and (80% to 90%) sensitive to clindamycin, erythromycin and ceftriaxone indicating the current value of these different antibiotics in the treatment and prophylaxis of GBS infection in Dar es Salaam.

## Conclusion

The high prevalence of GBS colonisation among pregnant women and neonates calls for screening of this bacterium in women attending antenatal care so that intrapartum antimicrobial prophylaxis can be offered to all women identified as carriers.

## Competing interests

The authors declare that they have no competing interests.

## Authors' contributions

AJ designed the study and supervised interviews, data collection and laboratory work. EFL and MIM assisted in design of the study and supervised laboratory work. FAM supervised clinical work. Finally, all authors participated in preparation of the manuscript, read and approved the final manuscript.

## Pre-publication history

The pre-publication history for this paper can be accessed here:

http://www.biomedcentral.com/1471-2458/9/437/prepub
